# Unintentional Retinal Displacement in Eyes Treated for Rhegmatogenous Retinal Detachment Complicated by Proliferative Vitreoretinopathy with Pars Plana Vitrectomy and Silicone Oil

**DOI:** 10.1155/2021/5532787

**Published:** 2021-05-22

**Authors:** Mariaelena Filippelli, Pasquale Napolitano, Ciro Costagliola, Michele Rinaldi, Flavia Chiosi, Roberto dell'Omo

**Affiliations:** ^1^Department of Medicine and Health Sciences “Vincenzo Tiberio”, University of Molise, Via Francesco De Sanctis 1, Campobasso 86100, Italy; ^2^Eye Clinic, Multidisciplinary Department of Medical,Surgical and Dental Sciences, University of Campania “Luigi Vanvitelli”, Naples 80131, Italy; ^3^Department of Ophthalmology, Azienda Ospedaliera dei Colli AORN Monaldi, Naples 80100, Italy

## Abstract

**Purpose:**

To examine the clinical characteristics, outcomes, and rate of unintentional displacement in eyes treated for rhegmatogenous retinal detachment (RRD) with pars plana vitrectomy (PPV) and silicone oil (SO).

**Methods:**

This retrospective observational study examined 50 eyes of 50 patients who underwent surgical repair for primary RRD complicated by proliferative vitreoretinopathy (PVR) by PPV and 1000-centistoke SO injection at a single institutional centre. The patients assumed a face-down posture immediately after surgery. Blue-fundus autofluorescence (B-FAF) pictures were obtained at 1 month after surgical procedures using a Spectralis HRA + OCT (Heidelberg Engineering, Heidelberg, Germany).

**Results:**

The primary success rate was obtained in 44 eyes (88%), on which the final analysis was conducted. Preoperative PVR was grade A in 7 eyes (15.9%), grade B in 28 eyes (63.6%), and grade C in 9 eyes (20.5%). The fovea was off and the detachment involved both the superior and inferior hemispheres of the retina in all cases. Breaks were located in the upper quadrants in 19 eyes (43.2%), in the lower quadrants in 12 eyes (27.3%), and in both upper and lower quadrants in 13 eyes (29.5%). The mean number of breaks was 3.4 ± 1.9. Intraoperative PFCL was used in 30 eyes (68.2%). Peeling of the epiretinal membrane/internal limiting membrane in the macula area was performed in 13 eyes (29.5%) during the first operation and carried out in all other eyes in occasion of SO removal. Preoperative BCVA was 2.1 ± 1.0 logMAR and improved to 0.8 ± 0.7 logMAR at the last follow-up (*P* < 0.0001). An upward unintentional retinal displacement was observed in 2 cases (4.5%).

**Conclusion:**

PPV and SO tamponade for complicated RRD are associated with good anatomical and functional outcomes and a very low rate of unintentional retinal displacement. Of the factors potentially implicated in favouring displacement that were studied, none were found significant.

## 1. Introduction

In recent years, the dramatic improvements in technology, instrumentation, and viewing systems have made pars plana vitrectomy (PPV) the preferred method for treatment of rhegmatogenous retinal detachment (RRD) by many ophthalmologists [1–3]. Repair of RRD with PPV is often accompanied by intraocular injection of gas or silicone oil (SO) at the end of the operation. It has been reported that SO is a safe and efficient tamponade for the treatment of primary uncomplicated RRD [4], but it is usually chosen over gas for traumatic cases, detachments associated with giant retinal tears (GRT), detachments complicated by proliferative vitreoretinopathy (PVR) [5, 6], or in the event that a postoperative airplane flight or high-elevation travel is planned [7, 8].

Both tamponades, SO and gas, in conjunction with PPV for the repair of RRD, may cause unintentional displacement of the retina, which is revealed by the presence of lines of increased autofluorescence in blue-fundus autofluorescence (B-FAF) imaging [9–11]. These lines are separated from the adjacent retinal vessels and run approximately parallel to them, closely reflecting their calibre and orientation. They have been termed “retinal vessel printings” (RVPs) by dell'Omo et al. [12] and “retinal pigment epithelium (RPE) vessel ghosts” by Lee et al. [10].

Previous studies have shown that unintentional retinal displacement occurs much more rarely in eyes filled with SO than in eyes filled with gas [9–11]. In the largest analysis reported in the literature, including 28 RRDs treated with PPV and 1000-centistoke (cst) SO, dell'Omo et al. [11] found retinal displacement in only 14.3% of the cases. At present, it has not been elucidated whether the severity of PVR, the presence of a macular hole, and additional surgical manoeuvres like the peeling of the macular epiretinal membrane (ERM) or internal limiting membrane (ILM) may influence the rate of displacement in eyes tamponated with SO. In the current series, we report the outcomes and the rate of unintentional displacement in eyes treated with PPV and SO to treat RRD complicated by different grades of PVR and by other clinical characteristics that put these eyes at high risk of developing redetachment.

## 2. Methods

This retrospective observational study included patients treated for fovea-off RRD with PPV and SO injection at a single tertiary referral centre (University of Molise, Campobasso, Italy) by a single surgeon between December 01, 2018, and June 30, 2020. All cases were complicated by PVR, which was defined as follows: grade A: vitreous haze and pigment; grade B: wrinkling of the inner retinal surface, retinal stiffness, rolled edge of breaks, and vessel tortuosity; and grade C: full-thickness and fixed retinal folds [13]. The reasons for injecting SO were the following: the presence of PVR C, hypotonus (defined as intraocular pressure ≤ 6 mmHg measured with applanation tonometry associated with choroidal detachment), giant retinal tear, 2 or more breaks located in the lower quadrants, multiple breaks located in the upper and lower quadrants, unidentified retinal breaks, macular hole associated with retinal detachment, and residency at an elevation above 1000 m.

The study was approved by the Institutional Review Board of the University of Molise and adhered to the tenets of the Declaration of Helsinki (1964). In view of the retrospective nature of the study and since all the procedures performed were part of the routine care, the Ethics Committee waived the need of collecting informed consent from participants.

Patients were excluded if they had a previous history of ophthalmic surgery (except for noncomplicated phacoemulsification), degenerative myopia (defined as the axial length of the globe ≥26.5 mm associated with degenerative changes in the sclera, choroid, and retinal pigment epithelium/retina [14]), glaucoma, wet age-related macular degeneration, ocular vascular diseases such as diabetic retinopathy and retinal vein occlusion, and gross macular ERM.

Before the operation, all the patients underwent a comprehensive ophthalmic examination including anterior segment examination, dilated funduscopy, Optical Coherence Tomography (OCT), B-FAF imaging, and measurement of the logarithm of the minimum angle of resolution (logMAR) best-corrected visual acuity (BCVA) using early treatment diabetic retinopathy study (ETDRS) charts at 4 meters. Visual acuity of counting fingers was converted to 1.4, that of hand movements was converted to 2.7, and that of light perception was converted to 3.7 logMAR. Preoperative files were consulted in order to retrieve the clinical characteristics of detachment (location, quadrants involved, location and number of retinal breaks, and grade of PVR), lens status, and time from the onset of symptoms suggestive of RRD to surgery.

If further breaks were noted intraoperatively, they were reported and considered in the final data analysis. A number of breaks equal to 1 were attributed to eyes in which no break was detected in either pre- or intraoperative examination. OCT scans and 50-degree B-FAF images (excitation wavelength at 488 nm and barrier filter at 500) were acquired with a Spectralis HRA + OCT (Heidelberg Engineering, Heidelberg, Germany). Images were taken at baseline and 1 month after surgical procedures (i.e., 1 month after PPV to repair the detachment and 1 month after SO removal). Two graders (MF, PN) independently assessed the presence of RVPs and their position (upward or downward) in relation to the adjacent retinal vessels. When there was disagreement between the two graders, a third grader (RdO) decided which grader's judgment should be accepted.

### 2.1. Surgical Procedure

All operations were performed after inducing local anaesthesia by peribulbar block. Phakic eyes underwent phacoemulsification and intraocular lens (IOL) implantation before starting PPV. Twenty-three-gauge PPV was performed in all cases with the constellation vitrectomy system (Alcon Labs, Fort Worth, TX). The surgical procedure involved separation and removal of the posterior hyaloid if not already detached, extensive, and meticulous vitrectomy with the shaving of the vitreous base, and relief of all vitreous tractions on retinal tears.

The epiretinal membrane and ILM were peeled from the macular area at the surgeon's discretion when the macular area appeared wrinkled based on preoperative OCT evaluation or during the intraoperative examination. Bimanual manoeuvres using forceps and scissors were used to grasp and peel preretinal membranes in correspondence with star folds in cases complicated by PVR C and to dissect the vitreous base. Neither retinectomies nor encircling scleral buckles were used in any case.

Perfluorocarbon liquid (PFCL) was used at the discretion of the surgeon. Retinopexy was achieved by laser endophotocoagulation or cryopexy. 1000-cst SO was used as the final tamponade in all cases. Injection of SO was preceded by a fluid-air exchange in some cases; otherwise, a direct PFCL/SO exchange was carried out. When air-fluid exchange was used, particular care was paid to drain as much subretinal fluid as possible from an existing break.

Immediately after the operation, the patients were asked to maintain a strict face-down posture for 24 hours, independently of the location of the breaks. All the eyes underwent a second surgical procedure to remove SO, 2 to 5 months after the primary surgery. All patients included in this study had a follow-up of at least 3 months after SO removal. Data were collected on ocular adverse events, such as the development of the redetachment following SO injection, macular oedema, intraocular pressure (IOP) ≥ 22 mmHg, band keratopathy, and acute vision loss.

### 2.2. Statistical Analysis

Descriptive statistics are presented as the mean ± standard deviation (SD). Comparison between pre- and postoperative BCVA was performed using the Wilcoxon signed-rank test. The relation between the occurrence of unintentional displacement and clinical characteristics of detachment was investigated using stepwise logistic regression analysis. The interrater agreement between graders for detection and position of RVPs relative to retinal vessels was determined with weighted *κ* statistics. Statistical analysis was performed using MedCalc version 11.5.1 (MedCalc software, Mariakerke, Belgium).

## 3. Results

Between December 1, 2018, and June 30, 2020, 91 eyes underwent PPV and SO injection to repair retinal detachment at the Department of Ophthalmology, University of Molise, Campobasso, Italy. Fourty-seven patients were excluded from the final analysis for the following reasons: inability to attend the follow-up visits (*n* = 4); traumatic retinal detachment (*n* = 6); detachment associated with proliferative diabetic retinopathy (*n* = 5), retinal vein occlusion (*n* = 2), degenerative myopia (*n* = 8), wet age-related macular degeneration (*n* = 3), and gross macular ERM (*n* = 3); and low-quality images of the retina (*n* = 10). Also excluded were 6 patients who developed redetachment during follow-up. Redetachments occurred in 4 eyes with preoperative PVR C and in 2 eyes with PVR B for the onset of new breaks; all redetachments occurred prior to SO removal. Thus, 44 eyes of 44 patients (29 males, 15 females) were included in the final analysis.

The reasons for using SO tamponade were the following: PVR grade C (*n* = 9), hypotonus (*n* = 2), giant retinal tear (*n* = 3), 2 or more breaks located in the lower quadrants (*n* = 12), breaks located in the upper and lower quadrants (*n* = 13), unidentified retinal breaks (*n* = 1), presence of a macular hole along with other peripheral breaks (*n* = 3), and residence at elevation above 1000 m (*n* = 1). The mean age at the time of operation was 65.8 ± 11.4 (range 23–87) years. The mean duration (±SD) of retinal detachment before the operation based on the self-reported symptoms was 15.8 ± 22.8 (range 1–120) days. At enrolment, 29 eyes (65.9%) were phakic and all underwent phacoemulsification and IOL implantation in combination with PPV.

The fovea was off and the detachment involved both the superior and inferior hemispheres of the retina in all cases. RRD involved three quadrants in seventeen eyes (38.6%) and four quadrants in 27 eyes (61.4%). Breaks were located in the upper quadrants in 19 eyes (43.2%), in the lower quadrants in 12 eyes (27.3%), and in both upper and lower quadrants in 13 eyes (29.5%). The mean number of breaks (excluding the eyes with GRT) was 3.4 ± 1.9. Three eyes presented with a macular hole (associated with a break in the lower retina in one case and with breaks in the upper and lower quadrants in 2 cases).

PVR was grade A in 7 (15.9%), grade B in 28 (63.6%), and grade C in 9 (20.5%) eyes. Peeling of ERM/ILM in the macula area was performed in 13 eyes (29.5%) at the time of RRD repair and carried out in all other eyes in the occasion of SO removal. PFCL was used intraoperatively to flatten the retina in 30 eyes (68.2%). At the end of the operation, a balanced salt solution (BSS) was exchanged with air and air with 1000-cst SO in 14 eyes (31.8%) eyes.

Among the eyes that received PFCL intraoperatively, in 17 cases (38.7%), a bleb of PFCL was left at the posterior pole when performing a fluid-air exchange with drainage of subretinal fluid from an existing peripheral break and removed after flattening of the peripheral retina. In the remaining 13 eyes (29.5%), a direct PFCL/SO exchange was carried out. The mean duration of SO tamponade was 2.04 ± 1.67 months (range 2–5 months).

During the follow-up period, the following complications were observed: a bleb of SO migrated in the anterior chamber in 2 eyes (4.5%), macular oedema developed in 3 eyes (6.8%), and an IOP ≥ 22 mmHg was recorded in 7 eyes (15.9%). The SO that migrated in the anterior chamber was removed at the time of SO removal from the vitreous cavity. Macula oedema and elevated IOP were treated successfully with topical prednisolone and antiglaucomatous drops, respectively. There were no cases of endophthalmitis; choroidal, subretinal, or vitreous haemorrhage; band keratopathy; or acute vision loss during the entire follow-up period.

The mean follow-up period after SO removal was 4.8 ± 2.7 months (range 3–9 months). In occasion of SO removal, ILM peeling at the macula was performed in all eyes where it had not been done during the first operation. Preoperative BCVA was 2.1 ± 1.0 logMAR and improved to 0.8 ± 0.7 logMAR at the last follow-up (*P* < 0.0001). Pre-, intra-, and postoperative characteristics of the sample are summarized in Table 1.

**Figure 1:**
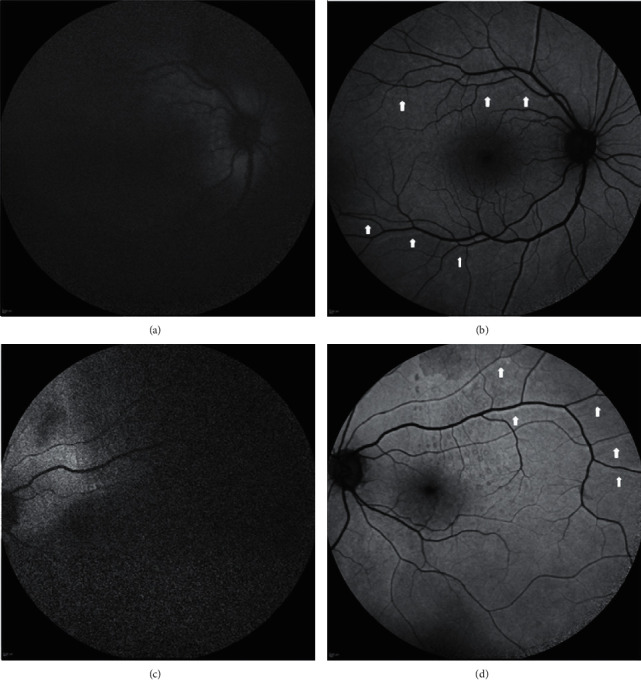
Unintentional displacement of the retina observed on blue-fundus autofluorescence (B-FAF) imaging 1 month after repair of fovea-off rhegmatogenous retinal detachment with PPV and 1000-centistoke silicone oil (SO) tamponade. Both eyes presented with preoperative proliferative vitreoretinopathy grade B received perfluorocarbon liquid intraoperatively, and a fluid-air exchange before injection of SO. Detachment involved 4 quadrants (with the breaks located in the upper and lower quadrants) in the case shown in (a) (the right eye of a 64-year-old lady) and 3 quadrants (with the breaks located in the upper quadrants) in the case shown in (c) (the left eye of a 74-year-old man). After retinal reattachment (b, d), retinal vessel printings (arrows) in the form of lines of increased autofluorescence running inferiorly to the corresponding retinal vessels were visible and indicated an upward displacement of the retina in both eyes.

One month after the first operation, RVPs located inferiorly to the retinal vessels (indicative of upward displacement) were noted in 2 eyes (4.5%) (Figure 1). Both of these eyes presented preoperatively with PVR grade B, and in both cases, PFCL was used intraoperatively to flatten the retina, and a fluid-air exchange was performed before injection of SO. Detachment involved 4 quadrants in one case (breaks located in the upper and lower quadrants) and 3 quadrants in the other (with the break located in the upper quadrants).

The regression analysis did not find factors significantly associated with the displacement (the independent variables put in the model were duration of detachment, detachment localization, quadrants involved, breaks location, breaks number, PFCL use, and type of exchange). Given that only 2 eyes showed displacement, the calculation of *κ* coefficient for the attribution of the presence of RVPs and their position relative to the retinal vessels was not feasible. Throughout follow-up, before SO removal, the calibre, orientation, and distance of RVPs from the adjacent retinal vessels remained stable. The presence and location of the RVPs in relation to the adjacent retinal vessels did not change after the removal of SO and ILM peeling.

## 4. Discussion

This study shows that unintentional displacement of the retina after repair of fovea-off RRD with PPV and SO is a rare complication that occurs in less than 5% of cases. The severity of PVR, extent of detachment, location of the breaks, use of PFCL, peeling of ERM/ILM, and modality of exchanging tamponades did not influence the occurrence of retinal displacement.

In the last decades, the advances in instrumentation technology and the possibility of monitoring the retina reattach intraoperatively have made PPV the preferred technique by the great majority of surgeons for the repair of complicated RRD [1–3]. In conjunction with PPV, gas or silicone oil tamponades are typically left in the eye at the end of the surgical procedure. Given their high interfacial tension and buoyancy force, gases, including air, are very efficient tamponades. However, SO is preferred by many ophthalmologists to treat retinal detachments complicated by PVR grade C or worse and those associated with giant tears, multiple tears involving both upper and lower quadrants, proliferative diabetic retinopathy, or trauma [6]. The use of SO is also indicated for patients living at high altitudes (>1000 m) because variations in atmospheric pressure associated with altitude can cause an expansion of the gas bubble and an acute increase in IOP, which can result in retinal vascular occlusions [7, 8].

Although SO is a helpful tool for managing severe retinal conditions, one main disadvantage associated with its use is the need for additional surgery to remove it. Several postoperative complications have been described following SO usage, such as cataract, increased IOP, and band keratopathy [15]. However, In the Silicone Study Report 2, which compared SO with gas tamponade in the treatment of RRD with PVR, the rate of complications, including elevated IOP and keratopathy, was not significantly different between eyes randomized to receive perfluoropropane (C_3_F_8_) gas and those randomized to receive SO [16]. Finally, SO in association with PPV may cause, albeit more rarely than gas, unintentional displacement of the retina, which in turn can cause metamorphopsia and strabismic deviation [10, 12, 17].

In 2010, Shiragami et al. [17] first reported the visualization of retinal displacement following repair of bullous and superior RRDs with PPV and gas. Using a fundus camera (FC) with special filters (530–580 nm excitation filter and 615–715 nm barrier filter), they were able to detect lines of increased autofluorescence running parallel to the retinal vessels. This revealed the anatomical position of the vessels before the detachment. In this seminal study, unintentional displacement was observed in 62.8% of patients. Subsequent series have found a variable incidence of these lines ranging from 14 to 72% [9–12, 17–19].

This variability may have resulted from several factors, including anatomical characteristics of the detachment, surgical techniques (including the type of tamponade used), posturing regimens adopted after the operation, and systems used to record images. In fact, (1) the displacement is likely to occur in eyes tamponated with gas and seems to be more marked when larger extents of the retina are detached [9, 10, 17] whereas it rarely occurs in eyes tamponated with SO [9–11]; (2) the adoption of face-down positioning immediately after operation effectively prevents retinal displacement [18]; and (3) FC may detect a marginally higher rate of retinal displacement compared to confocal scanning laser ophthalmoscopy (cSLO) [20].

In previous research, dell'Omo et al. [11] reported that the location of detachment, number of quadrants involved, and number or location of retinal breaks do not have a role in determining the displacement in eyes operated on with PPV and SO for uncomplicated RRDs or RRDs complicated by PVR grade A or B. However, to the best of our knowledge, no previous studies have evaluated whether PVR C, the presence of a macular hole, additional surgical manoeuvres like peeling of macular epiretinal membrane (ERM) or internal limiting membrane (ILM), and the modality of tamponade exchange may influence the rate of retinal slippage in eyes that have undergone PPV and SO tamponade.

In this study, we observed only 2 cases of retinal displacement, and no relationship was found with any of the variables used for the regression analysis. It must be noted that since all the cases of this series had 3 or 4 quadrants detached, it was not possible to assess whether the extent of retinal detachment was a risk factor for developing displacement, as observed in previous studies [10, 16]. We did not find RVPs in any of the eyes with macular hole, a condition in which subretinal fluid may be easily and quite thoroughly drained from the subretinal space but, quite surprisingly, we did not notice RVPs in the three eyes with GRT (despite GRTs being known risk factors for retinal slippage when gas is used as tamponade) [21, 22]. Finally, we did not find an increased proportion of displacement among the eyes with PVR C, despite the fact that retinal rigidity may potentially favour the reattachment of the retina in a site different from the original location.

Therefore, in keeping with previous observations [11], we believe that the low incidence of displacement reported in the current series must be correlated solely with the physical properties of SO. In fact, the relatively low interfacial tension (35 mN/m) and the much lower buoyancy force in comparison to gas [23] allow for SO bubbles to assume a spherical shape inside the eye (instead of the flat-bottomed shape assumed by a bubble of gas) and in an experimental eye model, Fawcett et al. estimated that an 80% fill of the vitreous chamber with SO would guarantee only a 180° angle of tamponade [24]. As a consequence, a bubble of SO exerts on the retina a force ∼30 times lower than that of a gas bubble of equal volume [25].

Thus, if the filling of the vitreous chamber does not approach 100%, many clock hours of the retina may remain uncovered. This in turn may allow the detached and stretched retinal surface to slowly return to its original size and location. Conversely, because of its physical properties, a gas bubble filling 80% of the vitreous chamber will guarantee a ∼300° angle of tamponade, forcing the detached retina against the eyewall before it may have the time to decrease and match its original location. This explains the results of previous studies all reporting a lower incidence of displacement occurring in eyes tamponated with SO in comparison to eyes tamponated with gas [9–11].

However, some considerations may be made regarding the low incidence of displacement reported in this study. It is possible that the quite long interval between the onset of detachment and the operation (mean 15 days) may have caused alterations of the underlying RPE, making the postoperative autofluorescent signal originating from the RPE (and thus the RVPs) less easy to detect. It is also possible that the use of a cSLO led to the underestimation of the presence of RVPs since it has been reported that cSLO-based B-FAF images may detect a marginally lower rate of vessel shift compared to FC [20].

In a recent multicentre retrospective consecutive case series that compared retinal displacement following RRD repair with pneumatic retinopexy (PR) versus PPV, Brosh et al. [26] found that the proportion of eyes that showed RVPs on B-FAF postoperatively was 7.0% for PR and 44.4% for PPV + gas (*P* < 0.001). Furthermore, the extent of the displacement was more severe in the eyes operated on with PPV than in those that underwent PR. The displacement was usually in the superior direction with PR and the inferior direction with PPV. The authors hypothesized that it is possible that the use of a small gas bubble (compared to a near 100% gas fill obtained during PPV), the concomitant presence of vitreous and gas, the slow reabsorption of subretinal fluid (compared with the forced internal drainage during PPV), and the different postoperative positioning may explain the low rate of displacement observed in the eyes treated with PR.

It is interesting to note that the bubble of gas used for PR or the bubble of SO injected at the end of PPV are in contact with an area of the retina smaller than that subtended by the bubble of gas filling the vitreous chamber after a near-complete air-fluid exchange. In fact, a partial filling (as in the case of PR) or a suboptimal filling (as in the case of SO tamponade) inevitably causes less displacement of subretinal fluid and, consequently, less displacement of the retina in comparison to a bubble of gas almost completely filling a vitrectomized eye. It remains to be ascertained why the displacement is directed upward following PR and in several eyes tamponated with SO, whereas it is preferentially downward in the eyes tamponated with gas. Larger studies may elucidate this aspect.

This study has some limitations. The first is that the sample analysed is relatively small. The second limitation is that this is a retrospective study with all the limitations inherent to such a design. Third, an agreed-upon “gold standard” for detecting vessel shift has not yet been established, so it is uncertain whether some displacements remained undetected in this study because of the methodology used to acquire B-FAF images or because we excluded poor-quality images prior to the grading.

The main strength of this study is the inclusion of eyes with different clinical characteristics operated on for detachment with PPV and SO by a single surgeon.

## 5. Conclusion

We found that PPV with SO injection for complicated RRD repair is associated with good anatomical and functional outcomes. Furthermore, the rate of unintentional retinal displacement is very low. Nevertheless, larger multicentre clinical trials are needed to consolidate and fully validate our results.

## Figures and Tables

**Table 1:** Main pre-, intra-, and postoperative characteristics of the sample.

Number of patients (eyes)	44
*Sex (M/F)*	29/15

*Age (years)*	65.8 ± 11.4

*Lens status at baseline, n eyes (%)*	
Phakic	29 (65.9)
IOL	15 (34.1)

*Detachment duration (days, mean ± SD)*	15.8 ± 22.8

*Quadrants of RRD, n eyes (%)*	
3	17 (38.6)
4	27 (61.4)

*Breaks location, n eyes (%)*	
Superior quadrants	19 (43.2)
Superior + inferior quadrants	13 (29.5)
Inferior quadrants	12 (27.3)

*Macular hole (in addition to peripheral breaks)*	3 (6.8)

*Breaks number/eyes (mean ± SD)*	3.4 ± 1.9

*PVR grade*	
A	7 (15.9)
B	28 (63.6)
C	9 (20.5)

*ERM/ILM peeling in the macular area at time of detachment repair*	
Yes	13 (29.5)
No	31 (70.5)

*PFCL use, n eyes (%)*	
Yes	30 (68.2)
No	14 (31.8)

*Exchange of tamponades*	
BSS/air/SO	14 (31.8)
BSS/PFCL-air/SO	17 (38.7)
BSS/PFCL/SO	13 (29.5)

*Duration of SO tamponade (months, mean ± SD)*	2.04 ± 1.67

*Duration of follow-up after SO removal (months, mean ± SD)*	4.8 ± 2.7

*Retinal displacement evidenced by RVPs*	
Present	2 (4.5)
Absent	42 (95.5)

*logMAR BCVA at baseline*	2.1 ± 1.0

*logMAR BCVA at last follow-up*	0.8 ± 0.7

M, male; F, female; IOL, intraocular lens; RRD, rhegmatogenous retinal detachment; PVR, proliferative vitreoretinopathy; ERM, epiretinal membrane; ILM, internal limiting membrane; PFCL; perfluorocarbon liquid; BSS, balanced salt solution; SO, silicon oil; RVPs, retinal vessel printings; logMAR, logarithm of the minimum angle of resolution; BCVA, best-corrected visual acuity.

## Data Availability

The datasets generated during and/or analysed during the current study are available from the corresponding author on reasonable request.
